# A Novel Genetic Neural Network Algorithm with Link Switches and Its Application in University Professional Course Evaluation

**DOI:** 10.1155/2022/9564443

**Published:** 2022-05-24

**Authors:** Honghai Ji, Jinyao Zhou, Shida Liu, Li Wang, Lingling Fan

**Affiliations:** ^1^Department of Electrical and Control Engineering, North China University of Technology, Beijing 100144, China; ^2^Department of Automation, Beijing Information Science and Technology University, Beijing 100192, China

## Abstract

This study exploits a novel enhanced genetic neural network algorithm with link switches (EGA-NNLS) to model the professional university course evaluating system. Various indices should be employed to evaluate the learning effect of a professional course comprehensively and objectively, and the traditional artificial evaluation methods cannot achieve this goal. The presented data-driven modeling method, EGA-NNLS, combines a neural network with link switches (NN-LS) with an enhanced genetic algorithm (EGA) and the Levenberg–Marquardt (LM) algorithm. It employs an optimized network structure combined with EGA and NN-LS to learn the relationships between the system's input and output from historical data and uses the network's gradient information via the LM algorithm. Compared with the traditional backpropagation neural network (BPNN), EGA-NNLS achieves a faster convergence speed and higher evaluation precision. In order to verify the efficiency of EGA-NNLS, it is applied to a collection of experimental data for modeling the professional university course evaluating system.

## 1. Introduction

Currently, the quality of colleges and universities is an essential issue. The learning effect of courses has become an important criterion for evaluating students' mastery of knowledge, and it also reflects teachers' teaching achievements and school management. However, the interference of objective and subjective factors in the real world makes it challenging to develop a mathematical model for evaluating the course effect. Some scholars in academia and education have presented their ideas on constructing an evaluation system for courses in colleges and universities in recent years. Meanwhile, some methods have been proposed for evaluating the learning effect of courses with specific attributes, including analytic hierarchy process, cluster analysis [1–3], fuzzy comprehensive evaluation method [4–8], and multiple regression analysis [9–12]. However, since different colleges and universities have different situations, a recognized and ideal learning effect evaluation system has not been constructed. Since it is challenging to obtain the course evaluation effect through a rigorous mathematical model, it is crucial to establish an objective, effective, and easy-to-operate course learning effect evaluation model.

Recently, artificial neural networks (ANNs) have grown quickly [13–15]. Since this method originates from the simulation of the brain nervous system, it has strong adaptability and self-learning ability in a complex environment. Meanwhile, another important feature of neural networks is that they can approximate each nonlinear continuous function with any precision and simulate actual systems realistically. The structure of neural networks can be regarded as the mapping of an actual system. Due to these characteristics, neural networks have been widely applied in various fields, including automatic control [16, 17], artificial intelligence [18], and fault diagnosis [19]. Different neural networks can be formed according to the neurons' topological structure. At present, the typical network models involve the backpropagation (BP), the perceptron, the radial basis function (RBF), the Hopfield, the Boltzmann machine, and the self-organizing networks. The mentioned network models can achieve various goals like pattern recognition, data clustering, function approximation, and optimization of computer prediction.

Currently, due to the prevalence of the BP neural network, as one of the widely used neural networks, the backpropagation neural networks (BPNN) learning approach has been utilized in most of the previous ANN literature. Nevertheless, the network structure is fixed in almost all of the previous studies. Such a fixed structure cannot provide neural network optimal performance. Although a large network may incur unnecessary implementation costs, it is challenging to obtain satisfactory accuracy by adopting a small network. For this reason, various techniques have been proposed for simultaneous optimization of the network's framework and connection weights, such as the quantum-based algorithm [20], improved genetic algorithm [21], a hybrid Taguchi-genetic algorithm [22], an evolutionary program called the generalized acquisition of recurrent links (GNARL) [23], the simulated annealing, and Tabu search algorithms [24].

Besides, the BP algorithm may fall into the local minimum, especially for large and irregular data sets. Genetic backpropagation neural networks (GA-BPNN) can be applied to solve this problem. GA-BPNN, which employs the parallel searching capability of genetic algorithms to improve the ability of BP neural networks in weight learning [25], has been widely utilized in the relevant literature [26–28].

This paper constructs the course learning effect evaluation model via an enhanced genetic BP neural network with link switches (EGA-NNLS), which depends only on historical I/O data during the evaluation process and significantly reduces the cost and complexity compared with the first principles-based modeling methods.

EGA-NNLS, which combines a neural network with link switches (NN-LS) with an enhanced genetic algorithm (EGA) and the Levenberg–Marquardt (LM) algorithm, exhibits the following properties:

1. The network's framework and connection weights can be adjusted simultaneously.
2. Compared with the standard GA (SGA), the proposed EGA subject to the course evaluating process characteristics can achieve a faster convergence rate.
3. It entirely employs the network's gradient information.

There are three stages while applying the EGA-NNLS. The first stage analyzes the influencing factors and the main contents of the evaluation system of the course learning effect in colleges and universities. The evaluation results are then quantified and divided into several grades. In the second stage, an initial NN-LS is built, which is learned and updated by EGA to simultaneously optimize the input-output relationship and the network framework of NN-LS. The following three enhancement approaches are employed to implement EGA: triple selection operation (TSO), which can protect high fitness individuals from being randomly varied by mutation operators and provide performance superior to the traditional “double selection” [29]; self-tuning crossover operation (STCO), which is extended from the typical arithmetic crossover operation, but more appropriate for the course evaluating problem; and the multispecies approach, which is utilized to solve untimely convergence problems partially. The third stage adopts the Levenberg–Marquardt (LM) algorithm to tune the partial weight connected network attained at the previous stage to utilize the network's gradient data.

The EGA-NNLS method avoids the impact of the experts' subjective factors in the traditional evaluation methods on the evaluation results and provides a general model of the course learning effect evaluation system. Therefore, this method has particular significance in theory and practice. The actual data of a university course is selected to construct the EGA-NNLS, and the efficiency of EGA-NNLS is demonstrated through the simulations.

The organization of the current paper is given in the following.  Section 2 briefly describes the problems of the evaluation system of the course learning effect. In Section 3, according to the characteristics of the evaluation system, the EGA-NNLS is constructed to investigate the course learning effect.  Section 4 simulates and analyzes the algorithm. In Section5, the conclusions of this paper are drawn.

## 2. Description of the Evaluation System of the Course Learning Effect

### 2.1. Connotation and Significance of the System

Theoretically, the evaluation of the course learning effect in colleges and universities shall make a comprehensive, objective, and fair judgment on the courses, teachers, and the students by using the theories, methods, and techniques of educational evaluation and teaching according to the policies, regulations, talent training objectives, and the school requirements. It provides valuable information for educational decision-making methods to maximize the development of courses with ideal effect. The effect evaluation system performs evaluation activities involving many contents and aspects. It is crucial for implementing the national education guidelines and policies and the microteaching management of the school and plays the following roles:

1. Stimulating teachers' enthusiasm and improving their quality.
2. The evaluation of the course learning effect is an essential means to stimulate teachers' teaching enthusiasm. It feeds back the scientific and objective evaluation results to teachers to play their strengths more actively and provide more knowledge, thinking enlightenment, and innovation guidance to students.
3. Improving the teaching quality.
4. Implementation of the teaching evaluation system according to modern theories and methods for the course learning effect evaluation can provide scientific standards for teachers' teaching quality and reference standards for students' acceptance of knowledge and the rationality of the courses. Teachers play an essential role in the teaching process, dialectically unified with students' dominant role in teaching. The learning effect evaluation can prompt teachers to clarify their teaching objectives and tasks and reflect and enhance the teaching impact and their personal teaching quality.
5. Strengthening the scientific construction and management of colleges and universities.
6. By evaluating the learning effect of each course, the structure, quality, and working conditions of the whole teachers can be understood. Also, it is possible to find out the problems and adjust the teachers' team pertinently. According to the scientific index system and evaluation standard for the course learning effect and the guiding ideology, principles, methods, and procedures of the modern education evaluation, objective and fair conclusions can be drawn to provide a reliable basis and objective standard for school leaders to improve the teaching staff structure and implement the objective management.

### 2.2. Description of the Problems of the Learning Effect Evaluation System

Some of the courses in colleges and universities are highly theoretical or practical. Since there are courses about tool use methods, ideology, and methodology, the evaluation indicators should be selected from the students' learning process. From the perspective of process management, multifactor interaction and multilinks are integrated into the whole teaching process. Thus, it is not easy to classify different disciplines and compare the learning effects of various courses, learning links, and learning objects.

Accordingly, the primary factors that can directly reflect the learning effect and have common features should be employed to design the evaluation system, enhancing the system's practical operability. The following elements are often employed in the existing course learning effect evaluation system in most practical cases:

1. Learning attitude.
2. Whether the learning is seriously taken, whether the preview is timely, and whether the homework is finished carefully.
3. Learning content.
4. Whether the difficulty of the content is appropriate, whether the content closely depends on the basic knowledge, and whether much cross knowledge is involved.
5. Learning ability.
6. Whether the students have a solid foundation of knowledge, the understanding ability, the ability to look for information, the practical ability, the ability to connect theory with practice, and the ability to understate all individual parts.
7. Learning methods.
8. Whether students can flexibly choose methods according to their own strengths, whether they pay attention to the cultivation of creativity, and whether they often consult teachers and interact with students.
9. Learning purpose.
10. Whether to master their own skills for the purpose, or to satisfy the requirements of teachers, parents, or examination.

**Figure 1:**
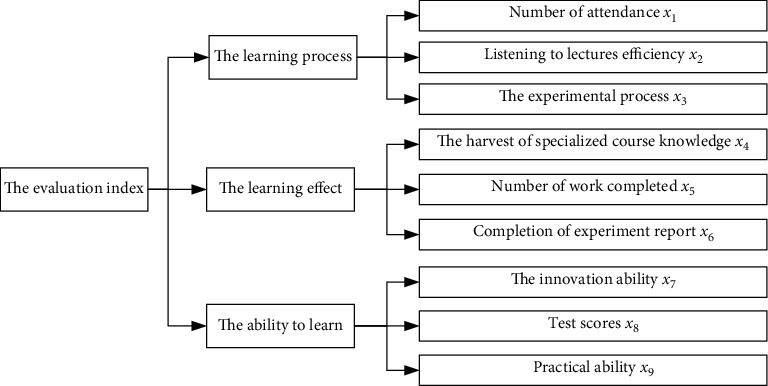
The evaluation index of course learning effect.

Based on the above five aspects, nine evaluation indices are obtained, as shown in Figure 1. The above nine evaluation indices are employed to study the measurement indexes in the proposed learning effect evaluation system.

## 3. Learning Effect Evaluation Model Using the Genetic Neural Network Algorithm with Link Switches

Based on the analysis in Section 2, the final learning effect of a course can be evaluated according to the comprehensive output results of the nine indices of *x*_1_–*x*_9_ determined through the learning process, effect, and ability, which can be described as follows:

(1) $$\begin{array}{c} I={\left[x_{1},x_{2},\ldots ,x_{9}\right]}^{T}. \end{array}$$

Meanwhile, the final evaluation levels can be divided into five levels of A–F, from good to bad. For the convenience of computer simulation, the five output levels are denoted as 3-bit binary codes.  Table 1 shows the correspondence between the levels and binary codes.

In this paper, the 3-bit binary number of the evaluation level is taken as the network output, written as

(2) $$\begin{array}{c} \hat{O}\left(k\right)={\left[y_{1},y_{2},y_{3}\right]}^{\prime }. \end{array}$$

Thus, the professional course evaluating process is represented as

(3) $$\begin{array}{c} \hat{O}=f\left(I\right), \end{array}$$

where *I* and $\hat{O}$ are denoted as equations (1) and (2), respectively.

The experts employ the traditional method according to the above indices. However, in some specific cases, the subjective factors of experts can affect the results of this evaluation method, leading to inaccurate judgment results. Therefore, the design of a data-based course evaluation system employs massive data to overcome the defects of conventional evaluation approaches and obtain more objective evaluation results.

In order to attain this goal, an EGA-NNLS method is presented. Through this method, it is not necessary to directly analyze the input-output relationship of the course evaluating model, while a data-driven approach is utilized for the modeling procedure.

EGA-NNLS comprises three essential components. As described in Section 3.1, the first component is an NN-LS, which can produce a partially connected network. The second component is an EGA, which is adopted to find the NN-LS's optimal weights. EGA is the generalized form of an SGA and will be described in Section 3.2. As the final component, the LM algorithm can further update the NN-LS, as illustrated in Section 3.3.

### 3.1. Building NN with Link Switches (NN-LS)

Conventional NNs generally have a fixed framework. Although an extensive network with many nodes and links may unnecessarily cause high implementation costs, a tiny network cannot provide satisfactory accuracy. Thus, a multi-input multi-output (MIMO) three-layer neural network with an adjustable number of links is employed.

**Figure 2:**
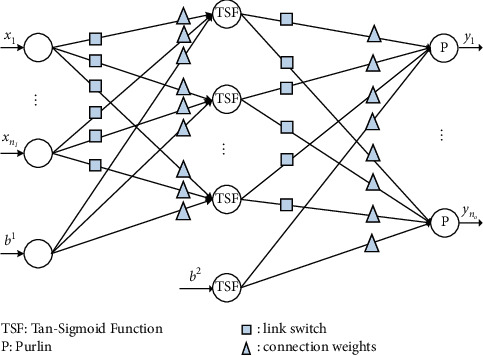
Framework of NN with link switches.

Figure 2 shows a standard network with link switches, in which a switch function can be described as

(4) $$\begin{array}{c} L\left(l\right)=\left\{\begin{array}{c} 0,\;l\leq 0;\\ 1,\;l>0. \end{array}\right. \end{array}$$

The connection weights of the NN-LS are described as follows:

(5) $$\begin{array}{c} w_{ij}^{1}=L\left(l_{ij}\right)\cdot r_{ij},\\ w_{jk}^{2}=L\left(l_{jk}\right)\cdot r_{jk}, \end{array}$$

where *i*=1,2,…, *n*_*I*_, *j*=1,2,…, *n*_*h*_, and *k*=1,2,…, *n*_*o*_, where *n*_*I*_, *n*_*h*_, and *n*_*o*_ stand for the number of input, hidden, and output nodes, respectively. *w*_*ij*_^1^ describes the connection weight between *i*‐th input and *j*‐th hidden nodes, and *w*_*jk*_^2^ stands for the connection weight between *j*‐th hidden and *k*‐th output nodes. *L*(*l*_*ij*_) and *L*(*l*_*jk*_) describe the link switches, demonstrating the lack or existence of the corresponding link. For an entirely connected network, in which all link switches are present, *w*_*ij*_^1^ and *w*_*jk*_^2^ will become the standard connection weights *r*_*ij*_ and *r*_*jk*_, respectively, similar to the corresponding ones in standard NN without link switches. For the university professional course evaluating process, *n*_*I*_ and *n*_*o*_ are chosen as 9 and 3, respectively.

The network's input and output vectors are denoted as equations (1) and (2), respectively. For the proposed NN-LS, the hidden layer output is described as

(6) $$\begin{array}{c} O_{\text{hidden}}\left(k\right)=\text{T}an‐\text{Sigmoid}\left(W^{1}\cdot I\left(k\right)+b^{1}\right). \end{array}$$

Based on Figure 2, the input-output relationship of the proposed NN-LS is given by

(7) $$\begin{array}{c} \hat{O}\left(k\right)=W^{2}\cdot O_{\text{hidden}}+b^{2}, \end{array}$$

where *W*^1^=[*w*_*ij*_^1^]_*i*=1,…,9, *j*=1,…,*n*_*h*__ stands for the weight matrix of the link between the input and hidden layers, *W*^2^=[*w*_*jk*_^2^]_*j*=1,…,*n*_*h*_,*k*=1,2,3_ stands for the weight matrix of the link between the hidden and output layers, *b*^1^=[*b*_*j*_^1^]_*j*=1,…,*n*_*h*__ and *b*^2^=[*b*_*k*_^2^]_*k*=1,…,*n*_*o*__ describe the biases for the hidden and output layers, respectively, and Tan‐Sigmoid(·) stands for the tangent sigmoid function defined as follows:

(8) $$\begin{array}{c} \text{Tan-Sigmoid}\left(x\right)=\frac{\left(1-e^{x}\right)}{\left(1+e^{x}\right)}. \end{array}$$

**Remark 1 .**

The transfer function of neural network mainly includes Purelin, Log-Sigmoid, and Tan-Sigmoid. Among them, Purelin is only applicable to the samples of linear mapping relationship. Compared with Purelin, Log-Sigmoid has a nonlinear mapping ability, but the gradient will disappear in the process of neural network backpropagation, and the output mean value cannot be zero. Tan-Sigmoid can avoid the problems of the above two functions, and it has a fast convergence speed and high precision. Therefore, equation (6) selects Tan-Sigmoid as the transfer function.

### 3.2. Adjusting the NN's Framework by EGA

Generally, the SGA can be applied to learn the network's input-output relationship in most cases. In the current study, the efficiency of SGA can be affected by too many optimal variables of the algorithm induced by the NN-LS's link switches and connection weights. Besides, since the course evaluation process's characteristics can reduce the diversity of individuals in the population, the premature problem can occur within the evolution process, reducing the SGA's performance.

For the mentioned reasons, EGA, an extension of the SGA, is proposed, which aims to effectively optimize the network framework and connection weight so as to ameliorate the premature problem.

To evaluate the algorithm performance, the following fitness function was selected:

(9) $$\begin{array}{c} \text{fitness}=\frac{1}{\text{MSE}}\text{,} \end{array}$$

where

(10) $$\begin{array}{c} \text{MSE}=\sum_{k=1}^{N_{tr}}\frac{{\left[\hat{O}\left(k\right)-O\left(k\right)\right]}^{T}\cdot \left[\hat{O}\left(k\right)-O\left(k\right)\right]}{N_{tr}}, \end{array}$$

where *N*_*tr*_ is the training set size and *O*(*k*) denotes the measured output at *k*‐th sampling time and is defined as

(11) $$\begin{array}{c} O\left(k\right)={\left[y_{1}\left(k\right),y_{2}\left(k\right),y_{3}\left(k\right)\right]}^{T}. \end{array}$$

For applying EGA, all the constructed NN-LS's connection weights and link switches presented in Figure 2 are transformed into the following chromosome:

(12) $$\begin{array}{c} \left[l_{ij}^{1},l_{jk}^{2},r_{ij}^{1},r_{jk}^{2},b_{j}^{1},b_{k}^{2}\right],\;i=1,\ldots ,n_{I},\;j=1,\ldots ,n_{h},k=1,\ldots ,n_{o}. \end{array}$$

Then, the EGA aims to find an optimal chromosome (12) to obtain the maximum fitness (9). And the following part 3.2.1–3.2.3 describes the three enhancement approaches adopted in the presented EGA.

**Remark 2 .**

The Mean Square Error (MSE) is a widely used evaluation index, which can avoid the problem that the positive and negative errors cannot be added together. In addition, because the error is squared, the role of the error with a large value in the index is increased, and the sensitivity is improved. Of course, other indicators can also be used, for example, Root Mean Square Error (RMSE), Mean Absolute Percentage Error (MAPE), and Symmetric Mean Absolute Percentage Error (SMAPE).

#### 3.2.1. Triple Selection Operation

Based on (12), the number of genes of a single chromosome, namely, the dimension of one possible solution in EGA, is calculated as

(13) $$\begin{array}{c} q_{\text{chr}}=2\times \left(n_{I}\times n_{h}+n_{h}\times n_{o}\right)+n_{h}+n_{I}. \end{array}$$

The number of genes in a single chromosome *q*_chr_, composed of all switches and connection weights, is relatively high even though there exist a few hidden nodes in the net. For instance, the length of a chromosome can reach 100 for *n*_*h*_ > 3. An individual's fitness can vary sensitively to the number of genes as *q*_chr_ grows. In such circumstances, the high fitness individuals are mostly chosen, but they also can be randomly varied through the mutation operation. In this regard, the first approach, named triple selection operation (TSO), can be employed to partially solve the problems induced due to the chromosome's high dimension.

**Figure 3:**
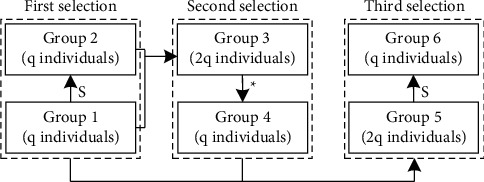
Schematic diagram of TSO. *S* stands for the Roulette selection, and *∗* stands for three operations, including selection, crossover, and mutation.

The schematic diagram of the triple selection operation is described in Figure 3, in which *q* stands for the population of the initial group. The first choice of all generations starts from Group 1 with initial *q* individuals. Group 2 is then formed by employing the standard “Roulette selection” approach with the reproduction probability as

(14) $$\begin{array}{c} \text{pro}b_{i}=\frac{f_{i}}{\sum f_{i}}, \end{array}$$

where *f*_*i*_ stands for the fitness of the *i*‐th chromosome. Group 3 incorporates Group 1 and Group 2 to generate Group 3 based on the second selection, crossover, and mutation operations. Rather than being taken as the final group, Group 4 is incorporated with the initial group again, and the third selection is then applied to the resultant group (Group 5). At last, Group 6 is achieved as the group employed in the next generation.

**Remark 3 .**

The proposed “double selection” approach [29] may not have enough ability to protect the high fitness individuals due to the high dimension of the studied chromosome. In “double selection,” Group 1 directly generates Group 4. However, an alternative selection operation is employed in TSO for forming Group 3 from Group 1. Compared with “double selection,” employing the additional selection for TSO can increase the probability of the final group inheriting superior genes. Besides, based on various experiments, the selection times are more than three, while the EGA's performance is hardly further enhanced, and the computational load will be increased.

#### 3.2.2. Self-Tuning Crossover Operation and Mutation

The selection operation is employed in all generations to determine the searching orientation direction toward the best individuals. Nevertheless, not any new individual is generated. In order to enhance the population's diversity, crossover operation is inevitably utilized in the genetic algorithm to interchange genes from the parents attained within the selection process. The crossover operation influences the selected population pairs and adds new individuals to the population according to the crossover rate *P*_*cv*_.

The second approach is called the self-tuning crossover operation (STCO), which is based on the standard arithmetic crossover operation and is more appropriate for the research in this paper. STCO creates three candidate offspring and takes two of them with the highest fitness as the final offspring by completing with each other. Accordingly, the parents' data can be entirely employed.

The following three steps are required to perform the STCO. Consider that each two selected parents are denoted by *P*_*x*_=[*g*_1_^*a*^, *g*_2_^*a*^,…,*g*_*q*_chr__^*a*^]^*T*^ and *P*_*y*_=[*g*_1_^*b*^, *g*_2_^*b*^,…,*g*_*q*_chr__^*b*^]^*T*^, where superscripts *a* and *b* stand for the indices of the parents to spread offspring.

**Step 1 C1.**

Calculate the following values:

(15) $$\begin{array}{c} G_{\text{max}}={\left[\max\left\{g_{1}^{1},\ldots ,g_{1}^{q}\right\},\ldots ,\max\left\{g_{q_{\text{chr}}}^{1},\ldots ,g_{q_{\text{chr}}}^{q}\right\}\right]}^{T},\\ G_{\min}={\left[\min\left\{g_{1}^{1},\ldots ,g_{1}^{q}\right\}\text{,}\ldots ,\min\left\{g_{q_{\text{chr}}}^{1},\ldots ,g_{q_{\text{chr}}}^{q}\right\}\right]}^{T}, \end{array}$$

where the superscript *q* is the number of the population. *G*_max_ and *G*_min_ stand for the two artificial chromosomes involving genes with maximum and minimum values within the total population of this generation, respectively.

For the chosen parents, obtain

(16) $$\begin{array}{c} P_{\max}={\left[\max\left\{g_{1}^{a},g_{1}^{b}\right\},\ldots ,\max\left\{g_{q_{\text{chr}}}^{a},g_{q_{\text{chr}}}^{b}\right\}\right]}^{T},\\ P_{\min}={\left[\min\left\{g_{1}^{a},g_{1}^{b}\right\},\ldots ,\min\left\{g_{q_{\text{chr}}}^{a},g_{q_{\text{chr}}}^{b}\right\}\right]}^{T}. \end{array}$$

**Step 2 C2.**

Create the following three children:

(17) $$\begin{array}{c} S_{1}=\alpha_{2}\cdot G_{\max}+\left(1-\alpha_{2}\right)\cdot P_{\max}={\left[g_{1}^{2},g_{2}^{2},\ldots ,g_{q_{\text{chr}}}^{2}\right]}^{T}, \end{array}$$

(18) $$\begin{array}{c} S_{2}=\alpha_{1}\cdot P_{x}+\left(1-\alpha_{1}\right)\cdot P_{y}={\left[g_{1}^{1},g_{2}^{1},\ldots ,g_{q_{\text{chr}}}^{1}\right]}^{T}, \end{array}$$

(19) $$\begin{array}{c} S_{3}=\alpha_{3}\cdot G_{\min}+\left(1-\alpha_{3}\right)\cdot P_{\min}={\left[g_{1}^{3},g_{2}^{3},\ldots ,g_{q_{\text{chr}}}^{3}\right]}^{T}, \end{array}$$

where crossover parameters *α*_1_, *α*_2_, *α*_3_ ∈ [0,1] are randomly chosen for each generation's evolution.

**Step 3 C3.**

Compute the fitness of *S*_1_, *S*_2_, and *S*_3_, and select two of them with the highest fitness as *S*_*a*_ and *S*_*b*_, respectively.

If *f*(*S*_*a*_) > *f*(*P*_*x*_) and *f*(*S*_*b*_) > *f*(*P*_*y*_), replace *P*_*x*_ and *P*_*y*_ with *S*_*a*_ and *S*_*b*_, respectively.

If *f*(*S*_*a*_) < *f*(*P*_*x*_) and *f*(*S*_*b*_) < *f*(*P*_*y*_), keep *P*_*x*_ and *P*_*y*_ unchanged.

Else, replace max{*f*(*S*_*a*_), *f*(*S*_*b*_)} with min{*f*(*P*_*x*_), *f*(*P*_*y*_)}.

**Figure 4:**
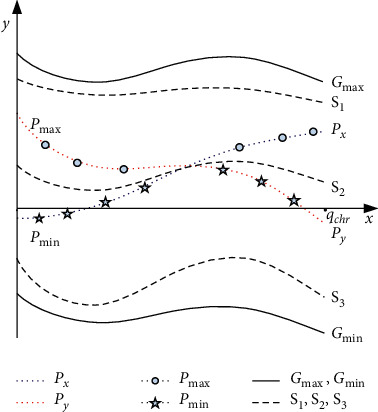
An illustrative example of STCO, the horizontal axis denotes the number of genes in a chromosome, and the vertical axis denotes the value of every gene.

Figure 4 presents an example to illustrate the concept of STCO, where *S*_2_ is obtained from *P*_*x*_ (blue line) and *P*_*y*_ (red line) using an approach similar to the standard arithmetic crossover operation. The main difference is that STCO sophisticatedly generalizes the parent group scope for crossover operation from *D*_2_=con*v*{*P*_*x*_, *P*_*y*_} to a broader domain spanned by *D*_1_=con*v*{*G*_max_, *P*_max_}, *D*_3_=con*v*{G_min_, *P*_min_}, and *D*_2_. In the sets *D*_1_ and *D*_3_, two more children, *S*_1_ and *S*_3_ are generated, as represented in (17) and (19), respectively. Thus, STCO more utilizes the parents' information compared with the standard arithmetic crossover operation. Finally, two offspring by STCO with the highest fitness, but not one by regular crossover operation, are selected.

After the crossover operation, the mutation operation will be applied to the population, by which the value of a gene of the population is randomly varied to incorporate new genetic materials into the population. The proposed EGA adopts the nonuniform mutation operation, which is widely utilized in genetic algorithms.

#### 3.2.3. Multiple Species, Migration, and Real Coding

The last improvement approach of the proposed EGA concentrates on dividing the population into various species. In most cases, the premature problem, which implicates a trade-off between exploration and exploitation, generally happens while employing SGA. The multiple species approach is utilized to solve the mentioned premature problem. By utilizing the mentioned approach, various species' search procedures can be applied simultaneously, and falling into local minimum can be avoided, which is generally induced by one species. Notably, three species are employed in the presented EGA. Specie 1 and Specie 2 perform the search individually by concentrating on a local but refined scope rather than broad scope. Specie 3 is utilized to merge Species 1 and 2 after completing the EGA.

The migration operation swaps individuals randomly between Species 1 and 2 in all generations. The migration rate *P*_*mr*_ adjusts the number of individuals moving between species. Generally speaking, a migration rate of about 5% per generation is a good choice.

There are various encoding algorithms like binary encoding and real encoding. The binary coding employs a simple on/off mechanism. Contrarily, applying binary encoding to the problems represented by real numbers may cause the “hamming cliff” phenomenon. In such circumstances, the real encoding should be employed just as we implement in the current paper.

#### 3.2.4. Layout of EGA and Benchmark Tests

**Figure 5:**
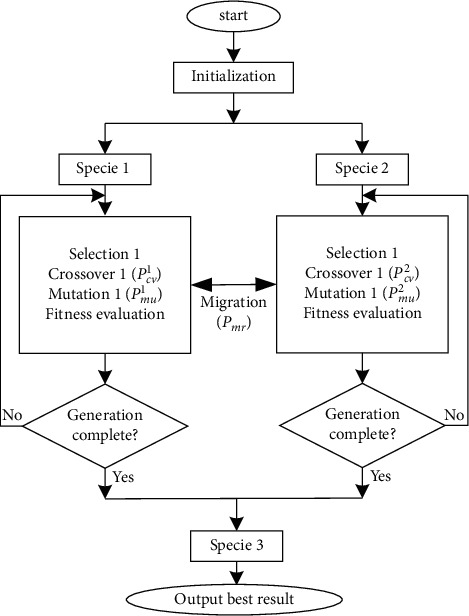
The layout of the proposed EGA with triple selection operation, self-tuning crossover operation, and three species.

Figure 5 describes the primary layout of the EGA. The fitness function (9) and the number of populations are employed to produce the initial population randomly. In virtue of the multiple species technique presented in the last part, the original population is randomly categorized into two species. The mentioned two species evolve separately using the presented TSO and STCO for a given number of generations. After that, the above two species are merged into Specie 3. Moreover, in this study, various crossover probabilities (described by *P*_*cv*_^1^ and *P*_*cv*_^2^) and mutation probabilities (described by *P*_*mu*_^1^ and *P*_*mu*_^2^ ) are adopted for two species in all generations.

The efficiency of the EGA is verified by four benchmark test functions to be applied in the next section. The benchmark functions are presented as follows:

Function 1:

(20) $$\begin{array}{c} f_{1}\left(x\right)=x\cdot \;\sin\left(10\pi x\right)+2,-1\leq x\leq 2, \end{array}$$

where the maximum value is at *f*_1_(1.8506)=3.8503.

The fitness function: fitness_1_(*x*)=*f*_1_(*x*).

Function 2 (De Jong function):

(21) $$\begin{array}{c} f_{2}\left(x\right)=\sum_{i=1}^{n}x_{i}^{2}\text{,}-5.12\leq x_{i}\leq 5.12, \end{array}$$

where *n* = 3 and the minimum value occurs at *f*_2_(0,0,0)=0

The fitness function: fitness_2_(*x*)=1/(1+*f*_2_(*x*)).

Function 3:

(22) $$\begin{array}{c} f_{3}\left(x\right)=\sum_{i=1}^{n-1}\left(100\right)\cdot \left(x_{i+1}-x_{i}^{2}\right)+{\left(x_{i}-1\right)}^{2}x_{i}^{2},\\ -2.048\leq x_{i}\leq 2.048, \end{array}$$

where *n* = 2 and the minimum value is at *f*_3_(0,0)=0.

The fitness function: fitness_3_(*x*)=1/(1+*f*_3_(*x*)).

Function 4 (Shubert function):

(23) $$\begin{array}{c} f_{4}\left(x_{1},x_{2}\right)=\sum_{i=1}^{n}\left(i\cdot \;\cos\left(\left(i+1\right)\cdot x_{1}+i\right)\right)\cdot \sum_{i=1}^{n}\left(i\cdot \;\cos\left(\left(i+1\right)\cdot x_{2}+i\right)\right),-10\leq x_{1},x_{2}\leq 10, \end{array}$$

where *n* = 2 and the minimum is at *f*_4_(−1.1432, −0.8003)=−186.7309.

The fitness function: fitness_4_(*x*_1_, *x*_2_)=−*f*_4_(*x*_1_, *x*_2_).

**Figure 6:**
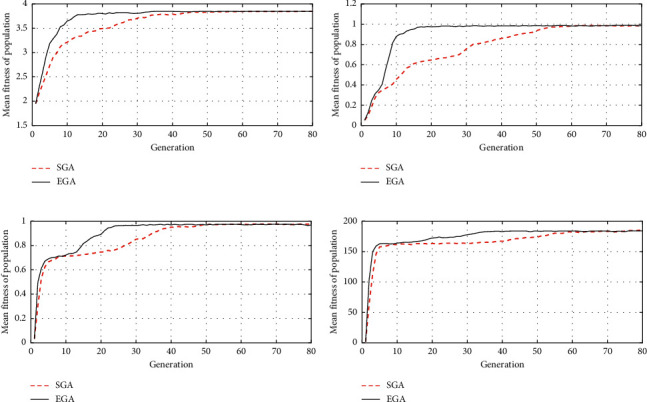
Average fitness of the population: SGA versus EGA. (a) Test function 1. (b) Test function 2. (c) Test function 3. (d) Test function 4.

The EGA employs the mentioned four test functions. The results are compared with those attained by the SGA with arithmetic crossover and nonuniform mutation. For every test function, the population size is 200 for EGA and SGA. The algorithm takes 80 iterations. The crossover probability is chosen as 0.6 for SGA and 0.6 and 0.8 for Species 1 and 2 in EGA. The mutation probability for functions is set at 0.04 for SGA and 0.04 and 0.06 for Species 1 and 2 in EGA.  Figure 6 presents the population's average fitness results obtained with EGA and SGA. Generally, it can be found that the population's average fitness value of EGA (solid black line) is better than that of SGA (red dashed line).

#### 3.2.5. Tuning the NN-LS's Framework and Connection Weights through EGA

After describing the EGA in part 3.2.1–3.2.4, the EGA can be used to adjust the proposed NN-LS framework and connection weights.

In each generation of EGA, the best chromosome of the contemporary population is chosen, and then all genes in this chromosome with the highest fitness through the population are derived. Specifically, the values of link switches *L*(*l*_*ij*_) and *L*(*l*_*jk*_) can be chosen either 0 or 1 depending on *l*_*ij*_ and *l*_*jk*_, which are available for each value of *i*, *j*, and *k*. Accordingly, the existence of *w*_*ij*_^1^ and *w*_*jk*_^2^ can be verified. In this regard, the framework of the NN-LS may change generation by generation to attain the optimum solution.

**Figure 7:**
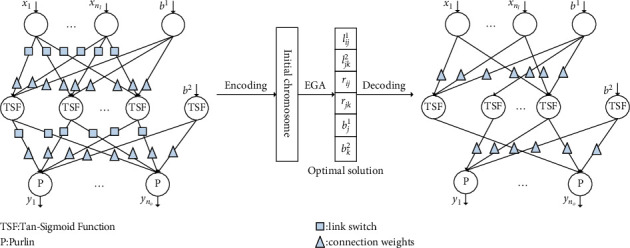
The topology of the EGA-NN-LS.

As presented in Figure 7, the original entirely connected feed-forward NN becomes a partially connected one after learning by EGA. Accordingly, the NN's implementation cost is reduced in terms of computing time.

### 3.3. Further Tuning the Connection Weights via the Levenberg–Marquardt Algorithm

As discussed in Subsection 3.2, the NN-LS framework and connection weights are tuned simultaneously by employing the presented EGA. Various performed tests indicate that higher accuracy can be obtained by NN-LS incorporated with EGA instead of the SGA. However, after EGA learning, the solution of maximum fitness function (9) may fall within the vicinity of the global minimum; therefore, in order to obtain the optimal solution, it is necessary to adopt a gradient-based algorithm to further update the weights in the neighborhood. The L-M algorithm is the most widely used algorithm with local search ability, which can effectively reduce the search conditions of GA and improve the stability of GA [30, 31]. Compared with other methods, the LM algorithm has faster convergence speed and accuracy and has been used to solve practical problems such as information physics system [32] and concrete compressive strength [33]. In order to attain higher efficiency, the (active) connection weights can be more tuned, as introduced in this subsection.

The steps of the L-M algorithm are listed in the following:

**Step 4 L1.**

Build a new connection weight vector as

(24) $$\begin{array}{c} X={\left[x_{1},x_{2},\ldots ,x_{m}\right]}^{T}. \end{array}$$

With EGA, the nonzero initial values *W*^1^, *b*_*j*_^1^,  *j*=1,…, *n*_*h*_, *W*^2^, and *b*_*k*_^2^, *k*=1,…, *n*_*o*_, can be obtained.

Calculate the following performance index, which evaluates the square error between the net's output and measured output,

(25) $$\begin{array}{c} R\left(X\right)=\sum_{k=1}^{N_{tr}}{\left[\hat{O}\left(k\right)-O\left(k\right)\right]}^{T}\cdot \left[\hat{O}\left(k\right)-O\left(k\right)\right], \end{array}$$

where *N*_*tr*_ stands for the size of the training set. *R*(·) can be regarded as a function with respect to the new weight vector *X* defined by (24). *O*(*k*) represents the training set's *k* − *th* measured output vector.

Here, updating the L-M algorithm is in batch mode. Define the following new error vector:

(26) $$\begin{array}{c} E={\left[e_{1,1},\ldots ,e_{n_{o},1},e_{1,2},\ldots ,e_{n_{o},2},\ldots ,e_{1,N_{tr}},\ldots ,e_{n_{o},N_{tr}}\right]}^{T}, \end{array}$$

where *e*_1,*j*_,…, *e*_*n*_*o*_,*j*_ denote all elements of $\hat{O}\left(j\right)-O\left(j\right)$.

**Step 5 L2.**

Noticing that the error vector *E* is also a function of *X*, calculate the Jacobian matrix as

(27) $$\begin{array}{c} J\left(X\right)≜\nabla E\left(X\right)=\left[\begin{array}{cccc} \frac{\partial e_{1,1}}{\partial x_{1}} & \frac{\partial e_{1,1}}{\partial x_{2}} & \ldots & \frac{\partial e_{1,1}}{\partial x_{m}}\\ \frac{\partial e_{2,1}}{\partial x_{1}} & \frac{\partial e_{2,1}}{\partial x_{2}} & \ldots & \frac{\partial e_{2,1}}{\partial x_{m}}\\ \vdots & \vdots & \vdots & \vdots \\ \frac{\partial e_{n_{o},N_{tr}}}{\partial x_{1}} & \frac{\partial e_{n_{o},N_{tr}}}{\partial x_{2}} & \ldots & \frac{\partial e_{n_{o},N_{tr}}}{\partial x_{m}} \end{array}\right]. \end{array}$$

Let

(28) $$\begin{array}{c} \Delta X_{k}=-{\left[J^{T}\left(X_{k}\right)\cdot J\left(X_{k}\right)+\mu_{k}\cdot I\right]}^{-1}\cdot J^{T}\left(X_{k}\right)\cdot E\left(X_{k}\right), \end{array}$$

where *μ*_*k*_ denotes a parameter to be tuned, and then update the connection weights *X*_*k*+1_ as

(29) $$\begin{array}{c} X_{k+1}=X_{k}+\Delta X\text{.} \end{array}$$

**Step 6 L3.**

Tune the parameters in (27) as follows:

If *R*(*X*_*k*+1_) ≤ *R*(*X*_*k*_), then *μ*_*k*_=*μ*_*k*_/*α*.

If *R*(*X*_*k*+1_) > *R*(*X*_*k*_), then *μ*_*k*_=*μ*_*k*_ · *α*, where *α* describes a positive constant.

**Step 7 L4.**

Stop if |*R*(*X*_*k*+1_) − *R*(*X*_*k*_)| < *ε*.

For calculation of *J*(*X*_*k*_) in Step L2, the chain rule can be employed, by which *X*_*k*+1_ can be updated by the standard BP method.

### 3.4. EGA-NNLS

By synthesizing NN-LS, EGA, and L-M algorithm in Subsections 3.1∼3.3, the following two stages can describe the EGA-NNLS algorithm:

**Stage 1 .**

Tuning the framework and weights of NN-LS simultaneously via EGA.

Calculate *n*_*I*_, *n*_*h*_, and *n*_*o*_ to construct a three-layer NN-LS. Then, all switches, weights, and bars within the NN-LS are mapped into a chromosome as (12) with the fitness function defined as (9). Accordingly, the initial population with a fixed number of individuals is randomly generated. The proposed EGA is then adopted to adjust the framework and weights of NN-LS simultaneously until converging the population's average fitness.

**Stage 2 .**

Further updating the nonzero connection weights.

When the first stage finishes, the structure of NN-LS is determined. In order to further search for the optimal nonzero weights, the L-M algorithm is applied.

**Remark 4 .**

For many problems such as the one discussed in (9), the nonzero connection weights tuned by EGA can be directly regarded as the final values of the network. However, the second stage is necessary for the studied problem, in which EGA-NNLS is incorporated with L-M.

## 4. Simulations

In order to verify the EGA-NNLS, this section performs simulations using the actual data of the evaluation effect of a particular university course. In the simulation process, the units of each evaluation index are unified, and the data are normalized to avoid the influence of data dimensions. This paper employs 40 groups of data samples, of which 30 groups are training set samples, and the other ten are the evaluation set samples.  Table 2 list the ten sets of test data to verify the effectiveness of the EGA-NNLS for predicting the learning effect. Based on the above data, EGA-NNLS is established, and the simulation parameters are listed in Table 3.

After setting the simulation parameters, EGA-NNLS is established. This paper takes a real number between −1.5 and 1.5 as the network output value. The binary code result can be obtained after rounding the absolute value of the network output result.

After completing the training of EGA-NNLS, the ten groups of test data listed in Table 2 are utilized to evaluate the efficiency of EGA-NNLS, and Table 4 lists the final results. It can be seen that the network output values after rounding are consistent with the actual binary code value in the ten groups of data for simulation.

**Figure 8:**
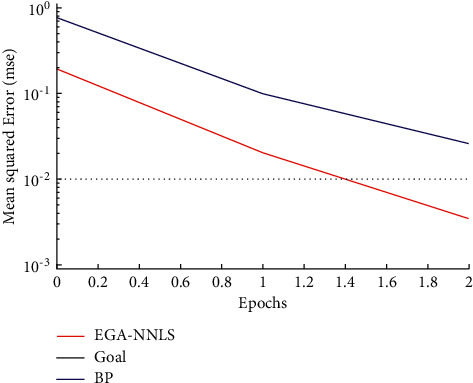
Comparing the performance function variations during training of the EGA-NNLS network with the BP neural network.

Figure 8 illustrates the performance function variations during the training process. Two weight updating algorithms are utilized in this paper. One is the proposed EGA-NNLS algorithm, and the other is the traditional BP algorithm. As shown in Figure 8, the performance functions of the two algorithms gradually decrease during the training process. However, compared with the conventional BP algorithm, the EGA-NNLS achieves the desired training accuracy after the second iteration (goal = 10^−2^, as shown in Table 3). Therefore, the superiority of the EGA-NNLS algorithm to the traditional BP algorithm is demonstrated in terms of efficiency and convergence speed.

It can be seen from the above simulation that EGA-NNLS can evaluate the learning effect. Compared with the traditional expert evaluation method, EGA-NNLS avoids the influence of subjective factors on the evaluation results. Meanwhile, the performance function of the EGA-NNLS network has a faster convergence speed in the training process than the conventional steepest descent BP neural network. Besides, EGA-NNLS can eliminate the adverse effect of gradient amplitude on the evaluation results in the network training process, achieving an accurate course evaluation effect. This demonstrates the excellent practicability of the proposed method.

## 5. Conclusions

The current work studies the modeling of the evaluation system of the course learning effect in colleges and universities. The existing evaluation system characteristics are employed to propose the EGA-NNLS evaluation and analysis system. A particular NN-LS is presented, and three enhancement approaches are utilized to combine it with the proposed EGA. Besides, as EGA utilizes the NN-LS's link switches, the LM algorithm further updates the network. Therefore, EGA-NNLS can learn the input-output relationships and the network framework simultaneously to model the course evaluation system, while the network gradient data is also entirely employed. Finally, the efficiency of EGA-NNLS is demonstrated through simulations of the actual data.

The proposed model is general, and the employed algorithm is based on the system data. Compared with the existing evaluation approaches like the analytic hierarchy process, fuzzy comprehensive evaluation approach, clustering method, and multiple regression analysis methods, the proposed model is mainly related to the data than the model mechanism. This demonstrates the efficiency and application value of this study.

## Figures and Tables

**Table 1:** The correspondence between the binary codes and the course evaluation effect levels.

Evaluation levels	Binary codes
A	001
B	010
C	011
D	100
E	101

**Table 2:** The test set data samples (10 groups).

The number of the test set samples										
	1	2	3	4	5	6	7	8	9	10
*x*_1_	0.77	0.80	0.85	0.60	0.75	0.72	0.57	0.80	0.85	0.60
*x*_2_	0.75	0.72	0.59	0.72	0.77	0.60	0.70	0.72	0.59	0.72
*x*_3_	0.82	0.93	0.63	0.67	0.82	0.60	0.62	0.93	0.63	0.67
*x*_4_	0.80	0.79	0.72	0.50	0.85	0.70	0.50	0.79	0.72	0.50
*x*_5_	0.94	0.68	0.82	0.58	0.92	0.80	0.64	0.68	0.82	0.58
*x*_6_	0.71	0.88	0.81	0.84	0.90	0.85	0.71	0.88	0.81	0.84
*x*_7_	0.87	0.67	0.77	0.60	0.85	0.71	0.57	0.67	0.77	0.60
*x*_8_	0.75	0.74	0.66	0.60	0.70	0.65	0.65	0.74	0.66	0.60
*x*_9_	0.83	0.81	0.74	0.59	0.81	0.70	0.53	0.81	0.74	0.59
Expert evaluation level	2	2	2	2	1	3	4	3	2	3
Corresponding code	010	010	010	010	001	011	100	011	010	011

**Table 3:** The simulation parameters of the EGA-NNLS.

Symbol	Value	Symbol	Value
*n*_*I*_	9	*N*_*tr*_	30
*n*_*h*_	8	*N*_*ts*_	10
*n*_*o*_	3	*P*_*cv*_^1^	0.7
Goal	10^−2^	*P*_*cv*_^2^	0.8
Epochs	300	*P*_*mu*_^1^	0.04
Population	200	*P*_*mu*_^2^	0.05
Generation	250	*P*_*mr*_	0.05
*α*	0.5		

**Table 4:** Comparison of prediction results and actual values of ten groups of the test set samples by EGA-NNLS.

Sample number	Network output code value			Value after rounding	Real code value
1	0.3706	0.9222	0.2093	010	010
2	−0.1556	1.3371	−0.8785	011	011
3	0.3856	0.7784	0.3343	010	010
4	0.0652	1.2600	−0.2607	010	010
5	−0.1387	1.2444	−0.7713	011	011
6	−0.3394	1.0860	−0.6106	011	011
7	0.992	0.38062	0.2093	100	100
8	−0.1756	1.3871	−0.9085	011	011
9	0.3856	0.7784	0.3243	010	010
10	0.0652	1.2600	−0.2607	010	010

## Data Availability

The data used to support the findings of this study are included within the paper.
